# Determinants of successful driving rehabilitation training in licensed individuals with disabilities

**DOI:** 10.1371/journal.pone.0322016

**Published:** 2025-04-24

**Authors:** Yun-Ji Jeong, Jun-Su Choi, Min-Ye Jung, Jung-Ran Kim, Yoo-Gyeong Jeong, Kyoung-Young Park, Myung-Ja Kong, Kwang-Soo Lee

**Affiliations:** 1 Department of Health Administration, Yonsei University Graduate School, Wonju, Gangwon-do, Republic of Korea; 2 Department of Occupational Therapy, Yonsei University Graduate School, Wonju, Gangwon-do, Republic of Korea; 3 Department of Occupational Therapy, College of Software and Digital Healthcare Convergence, Yonsei University,; 4 Department of Dementia Prevention and Rehabilitation, Catholic Kwandong University, Gangneung, Gangwon-do, Republic of Korea; 5 Department of Occupational Therapy, Jungwon University, Goesan County, Chungcheongbuk-do, Republic of Korea; National Institute of Technology Calicut, INDIA

## Abstract

Previous studies have provided that self-driving can enhance the mobility of people with disabilities and their quality of life. The National Rehabilitation Center has been providing driving rehabilitation education for people with disabilities since 1994, as part of a welfare service project aimed at guaranteeing their right to free movement. However, there is no analysis of the status and results of driving rehabilitation education and evaluations in South Korea, and research on these programs is lacking. This study aims to analyze the on-road driving rehabilitation education and evaluation results conducted by the National Rehabilitation Center from 2019 to 2021. It seeks to identify the characteristics of the prior license holders with disabilities and the factors influencing the need for additional driving rehabilitation education. Out of a total of 676 prior license holders, 532 were included in the analysis regarding the need for additional driving rehabilitation education. The results of this study indicate that women were 2.07 times more likely than men to require additional driving rehabilitation education. Conversely, the likelihood of requiring additional driving rehabilitation education was lower for those with better driving senses (0.17 times), less tension (0.46 times), and less impact from their disability (0.45 times). For prior license holders, it was found that demographic characteristics (excluding gender) or the nature of their disabilities had less significant impacts compared to the driver’s response level, the type of driving license held, and the number of assistive devices used. These findings can be used for developing effective driving education programs for people with disabilities and designing strategies to enhance license acquisition rates, thereby improving their mobility rights.

## Introduction

Several studies have established that driving is a crucial activity that enhances mobility and contributes to meaningful life [1–4]. In the Republic of Korea, the Act on the Promotion of Transportation for Mobility-Disadvantaged Persons ensures the mobility rights of individuals with disabilities and provides regulations for supporting driving in this population [5]. Despite these measures, many still face significant challenges in terms of mobility, including walking and using public transportation [6].

Research on the risks associated with driving by individuals with disabilities has revealed that the rates of collisions and traffic violations among drivers with disabilities are comparable to those in the general population [7–11]. However, individuals with acquired disabilities and those who have not driven for a prolonged period after obtaining a driver’s license may require additional practice and driving rehabilitation [4,12,13]. Moreover, individuals with disabilities who have undergone driving rehabilitation training are more likely to resume driving, which can facilitate social participation and enhance their quality of life [14,15].

The Ministry of Health and Welfare (MOHW) is responsible for overseeing disability welfare policies in accordance with the Act on the Welfare of Persons with Disabilities [16]. The National Rehabilitation Center (NRC), an entity under the MOHW, has been providing driving rehabilitation training for individuals with disabilities since 1994, as part of an initiative aimed at ensuring freedom of movement for individuals with disabilities. From 1994 to October 2022, the NRC conducted 13,199 rehabilitative training sessions. As of 2022, the program was operated by a team comprising nine driving rehabilitation instructors, one assistive technologist, one social worker, one occupational therapist, and two driving instructors.

The driving rehabilitation training program is divided into courses to obtain a driver’s license, and adaptation training for existing license holders. At present, both types of training at the NRC involve on-road driving instruction and evaluations conducted by driving instructors [5]. Off-road driving instruction and evaluation focus on various cognitive functions, such as executive functioning and self-awareness, and simulator assessments are used to replicate real driving environments for education and evaluation. However, these assessments may not accurately predict the ability to safely drive in real-life scenarios [13]. Driving is a complex process that integrates physical, sensory, attentional, and cognitive functions. Therefore, on-road driving education and evaluation are more reliable indicators of actual driving than off-road education and evaluation, and on-road training and evaluation have been found to be beneficial for individuals with disabilities in real-world driving environments [15,17].

There has been no comprehensive analysis of the driving rehabilitation training provided by the NRC since 1994, and an analysis of the outcomes of driving rehabilitation training and evaluation and research on the current state of driving rehabilitation programs in South Korea is limited [18]. It is crucial to analyze the status of individuals with disabilities who have received driving training and the outcomes of driving rehabilitation provided by the institution that has provided the longest-standing driving rehabilitation program in South Korea to further advance driving rehabilitation in the country [18].

Therefore, this study purposed to analyze the data from on-road driving rehabilitation and evaluation conducted as part of the driving adaptation project at the NRC from 2019 to 2021. Through this analysis, we seek to understand the general characteristics of previous driver’s license holders with disabilities who have participated in driving rehabilitation and evaluation at the NRC, and to identify the predictors of additional driving rehabilitation training.

## Materials and methods

### Study model

**Fig 1:**
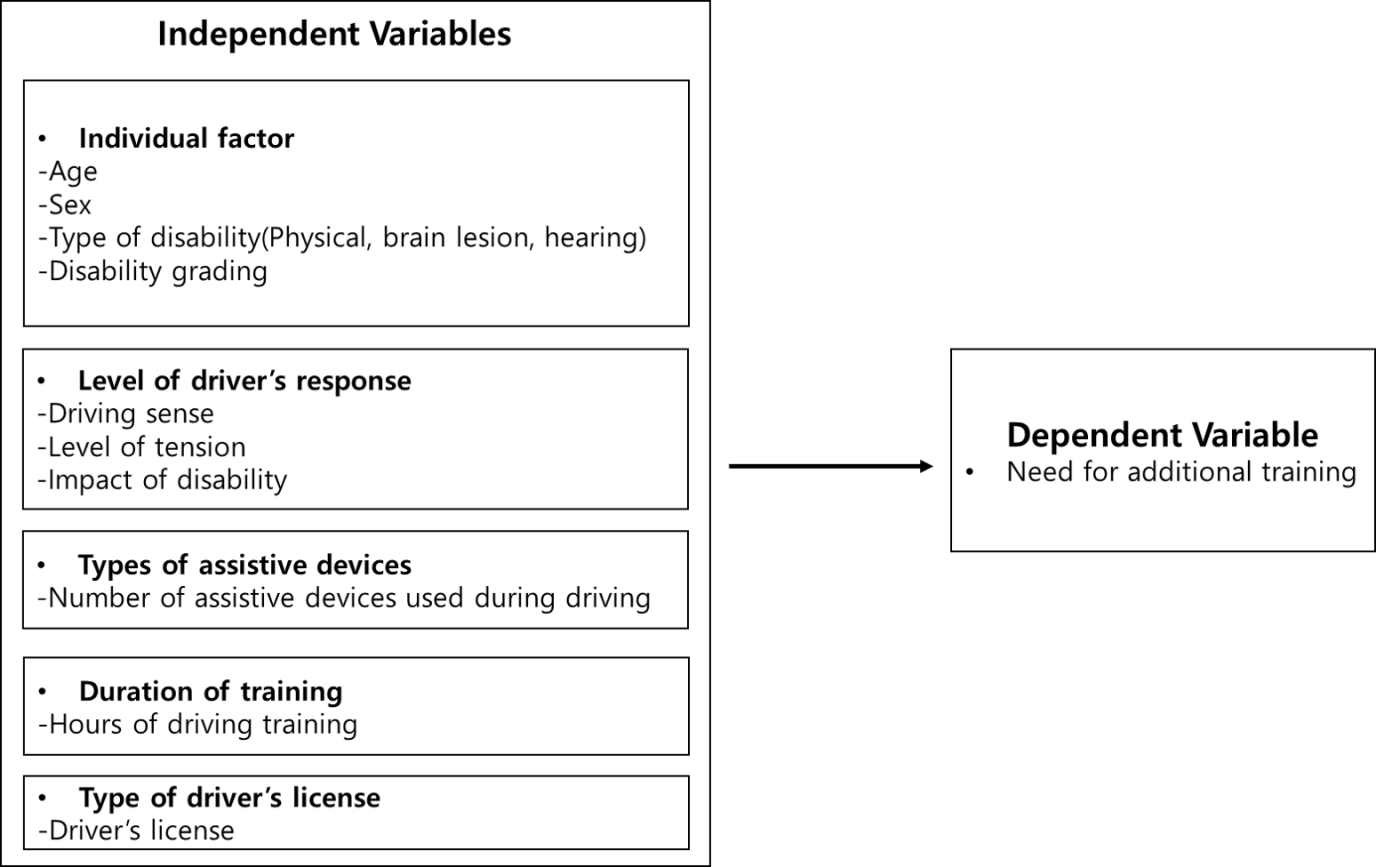
Analytical model for driver’s license holders.

In this study, we established the analytical model (Fig 1) with reference to the elements of the model proposed by Rajesh, R., Srinath, R., Sasikumar, R., & Subin, B. (2017) to identify the factors affecting driving [19].

### Study data and participants

The 2019–2021 NRC data on Driving Training and Evaluation Results for Individuals with Disabilities was used to assess the current need for supplementary driving rehabilitation among existing driver’s license holders and to identify key predictors influencing this need. For this study, we collected data from individuals who had participated in the NRC driving rehabilitation program in the past three years (2019–2021) and received permission from the institution for the use of the data. The requirement for informed consent was waived by the IRB owing to the non-invasive and retrospective study design. All personal information of the participants was de-identified before the data was provided to the authors. General characteristics included age, sex, disability type, disability grading, and driver response level (driving skills, tension, and influence of disability) rated by the driving instructor.

To analyze the need for additional driving rehabilitation training among existing driver’s license holders, data from 532 participants were included, after excluding data from 83 who did not answer the question about the existing driver’s license, 47 with two or more disabilities, and 14 with a heavy vehicle driver’s license. Including individuals with several disabilities may lead to confusion due to the interaction between the disabilities, and thus cloud the understanding of the impact of each disability. By limiting the study population to those with a single disability, we attempted to reduce the complexity of the study and facilitate a clear interpretation of the results. Furthermore, focusing on individuals with single disabilities may provide valuable insights into the specific educational needs of this population. This study was approved by the Institutional Review Board of Yonsei University Mirae Campus (IRB No: 1041849–202208-SB-152–01).

### Study variables

#### Dependent variables.

The dependent variable in the study was the requirement for additional driving rehabilitation training. This was determined based on a professional instructor’s subjective rating of the driver, that is, the “comprehensive rating” documented following the driving training for individuals with disabilities between 2019 and 2021. The driving instructor assessed the participants’ future driving abilities using established evaluation criteria. This assessment identified cases where participants were not yet ready to drive independently based on the training they had received, highlighting the need for additional instruction. The responses were divided into “able to drive without additional training” (0) and “requires additional driving training” (1).

Additional driving instruction refers to supplementary training sessions designed to improve the participant’s driving skills and overall readiness. These sessions follow a similar structure and content as the initial training but focus on providing extra practice and reinforcement in areas where improvement is needed.

#### Independent variables.

The independent variables included individual factor, driver response levels, vehicle interior features, training duration, and type of driver’s license [19]. Individual factor included sex, age, type of disability, and grade of disability. Patients were divided into male (0) and female (1) groups. Age was used as the continuous variable. The type of disability was divided into physical, brain lesion, and hearing disabilities according to the classification specified in the NRC driving training form and was dummy-coded. The NRC defines individuals with physical disabilities as those with permanent impairments in the limbs or torso, significant loss of hand or foot function, dwarfism, severe spinal deformities, or disabilities exceeding these conditions. Individuals with brain lesions are defined as having physical disabilities resulting from conditions such as cerebral palsy, traumatic brain injury, or stroke. Additionally, individuals with hearing impairments are characterized by severe hearing loss or significant balance disorders. Disability grading was divided into severe or grades 1–3 (0), and mild or grades 4–6 (1).

Types of assistive devices included driving assistive devices including left-hand steering rods, right-hand steering rods, left-hand controllers, right-hand controllers, left-side accelerator pedals, right-side turn signals, transfer boards, and extended pedals. The number of driving assistive devices currently in use by the participant was calculated and treated as a continuous variable. Although the program offered a total of eight assistive devices, each participant used a maximum of three devices. Therefore, for modeling purposes, the range of devices was defined as 0–3 to accurately reflect the participants’ actual usage.

Driver’s response level included driving skills, tension, and the influence of disability after driving training. All these variables were treated as continuous variables. The driving instructor rated the participant’s driving skill, tension level, and influence of disability after the training. Driving skills refer to a participant’s ability to understand and acquire driving-related knowledge and techniques. Scores range from 1 to 5, with 1 indicating “very poor” and 5 indicating “very good.” Tension measures the level of nervousness or stress experienced by the participant during driving training. A score of 1 represents “very tense,” while a score of 5 signifies “completely relaxed” or “no tension.” The influence of disability assesses how much the participant’s disability affects their driving training. A score of 1 indicates “very severe impact,” and a score of 5 reflects “no impact.”

The duration of training refers to the total time allocated for driving instruction. The NRC’s driving training program spans 4–5 days, with a total of 10 hours of training. It was categorized as ≤ 8 hours (0) or > 8 hours (1).

In this study, driver’s licenses were categorized into Class 1 (manual and automatic) (1) and Class 2 (manual and automatic) (0). Class 1 licenses allow drivers to operate larger vehicles, while Class 2 licenses are for smaller vehicles. The distinction between manual and automatic transmission is only related to the drivetrain mechanism and does not affect the range of vehicles a driver can operate, therefore the licenses were grouped by class without differentiating transmission type.

### Statistical analysis

The following analyses were performed:

First, participants’ general characteristics were analyzed using descriptive statistics.

Second, the effects of participants’ personal characteristics, number of assistive devices used, driver’s response level, duration of total driving training, and type of driver’s license on the need for additional driving rehabilitation were analyzed using binomial logistic regression. Factors associated with the need for additional driving rehabilitation were identified using logistic regression, and the relationships between these factors and the need for additional driving training were determined based on odds ratios (ORs). The model fit and accuracy of classification were tested using the Hosmer-Lemeshow test and C statistic, respectively [20–23]. Data were analyzed using SAS software version 9.4(SAS Institute Inc., Cary, NC, USA).

## Results

### General characteristics

Table 1 shows the general characteristics of the prior license holders included in the analysis for the need for additional driving rehabilitation. 532 were included in the analysis. Of them, 236 required additional training. The mean age of the participants was 44.89 years, and the most common type of disability was physical, followed by brain lesions and hearing impairment. In terms of grading, more individuals had severe disabilities. There were more people who had more than eight hours of driving training, and among the prior driver license holders, there were more people with Class 2 license.

**Table 1:** Descriptive statistics of prior license holders (N=532).

Variable			Frequency(%)	Mean(Standard deviation)
Dependent variable	Need for additional training	Yes	236(44.36)	
		No	296(55.64)	
Independent variable	Individual factor	Age		44.89(12.94)
		18≤N<20	6(1.13)	
		20≤N<30	76(14.29)	
		30≤N<40	90(16.92)	
		40≤N<50	137(25.75)	
		50≤N<60	156(29.32)	
		60≤N	67(12.59)	
		Sex		
		Male	259(48.68)	
		Female	273(51.32)	
		Type of disability		
		Physical	321(60.34)	
		Brain lesion	136(25.56)	
		Hearing	75(14.10)	
		Disability grading		
		Severe(grades 1~3)	380(71.43)	
		Mild(grades 4~6)	152(28.57)	
	Level of driver’s response	Driving sense		3.62(0.92)
		Level of tension		3.45(0.86)
		Impact of disability		3.39(0.82)
	Vehicle interior feature(Number of assistive devices used during driving)	0	342(64.29)	
		1	83(15.60)	
		2	94(17.67)	
		3	13(2.44)	
	Hours of driving training	Less than 8 hours	50(9.40)	
		Over 8 hours	482(90.60)	
	Type of driver’s license	Class 1	182(34.21)	
		Class 2	350(65.79)	

Binomial logistic regression was performed to analyze the predictors of the need for additional driving rehabilitation(Table 2). Sex, driving skill, tension, influence of disability, number of driving assistive devices used, and type of driver’s license were identified as significant predictors of the need for additional driving rehabilitation. The odds ratio for requiring additional driving rehabilitation were higher among women (2.07 times) than men, while the odds ratio for requiring additional driving rehabilitation were lower among those with better driving skills (0.17 times), those without tension (0.46 times), and those less influenced by disability (0.45 times). The accuracy of the classification of the study model was high (c=0.923), and the model fit (Hosmer-Lemeshow test) was good (χ2=4.260, p-value=0.833).

**Table 2:** Need for additional driving rehabilitation among prior license holders- results of logistic regression.

Variable		OR(95% CI)	p-value
Individual factor	Age	1.01(0.99-1.04)	0.206
	Sex(ref.: Male)		
	Female	2.07(1.14-3.77)	0.017
	Type of disability(ref.: Physical)		
	Brain lesion	0.79(0.40-1.56)	0.503
	Hearing	0.68(0.30-1.53)	0.352
	Disability grading(ref.: Severe(grades 1~3))		
	Mild(grades 4~6)	0.69(0.37-1.26)	0.228
Level of driver’s response	Driving sense	0.17(0.10-0.28)	<.0001
	Level of tension	0.46(0.26-0.80)	0.006
	Impact of disability	0.53(0.33-0.86)	0.010
Vehicle interior feature	Number of assistive devices used during driving	0.67(0.47-0.94)	0.021
Duration of training	Hours of driving training(ref.: less than 8 hours)		
	Over 8 hours	1.07(0.40-2.90)	0.901
Type of driver’s license	Driver’s license(ref.: Class 2)		
	Class 1	0.45(0.24-0.86)	0.015

CI: Confidence Interval, OR: Odds ratio

* -2 Log Likelihood: 730.727;

** Hosmer and Lemeshow goodness of fit test: χ^2^=4.260, p-value: 0.833;

*** c statistics: 0.923;

## Discussion

This study analyzed the characteristics of individuals with disabilities with a driver’s license who participated in driving rehabilitation at the NRC from 2019 to 2021. 532 were included in the analysis of the need for additional driving rehabilitation. The most common age group was 50–59 years, followed by 40–49 years. This differs from national disability statistics, which show the highest distribution of individuals with disabilities in the age group of 60 years and above [24]. This discrepancy may be attributed to factors related to disability type, social participation, and employment that affect participation in driving rehabilitation and training [24]. Among those who participated in driving training, the most common type of disability was physical disability (60.34%), followed by brain injuries (25.56%) and hearing impairments (14.10%). This also differed from the 2021 statistics for individuals with disabilities in Korea, which showed 64.35% with physical disabilities, 22.24% with hearing impairments, and 13.41% with brain injuries [24]. The discrepancy between the overall percentage of individuals with disabilities and those participating in driving rehabilitation can be explained as follows. Individuals with physical and brain injuries receiving inpatient or outpatient treatment at a rehabilitation hospital within the NRC participate in driving rehabilitation based on recommendations from occupational therapists. Therefore, the NRC should promote driving rehabilitation programs nationwide to ensure that more individuals with disabilities can benefit from the program. The number of individuals with disabilities who received driving rehabilitation between 2019 and 2021 included in this study is significantly lower than the overall number of individuals with disabilities. Thus, more proactive participant selection and customized driving rehabilitation training tailored to individual characteristics are needed to increase participation in driving rehabilitation among individuals with disabilities [18].

This study employed binary logistic regression analysis to examine the factors influencing the need for additional driving rehabilitation training. The dependent variable was binary, categorized into two distinct groups: “able to drive without additional training (0)” and “requires additional driving training (1).” Logistic regression, a method specifically designed for predicting binary outcomes, was deemed appropriate for this analysis. Furthermore, references to prior studies that successfully utilized logistic regression in similar contexts support the validity and robustness of the chosen methodology [20–23].

Data on the need for additional training were collected by driving instructors providing driving training to individuals with disabilities. The analysis revealed that sex, driving skill, tension level, influence of disability, number of assistive devices used while driving, and type of driver’s license held were significant predictors.

In our study, the odds ratio of requiring additional driving rehabilitation were higher among women than among men (2.07 times). This is consistent with previous findings, suggesting that female drivers require more attention and concentration during driving because of poorer spatial perception than their male counterparts [25]. Furthermore, the odds ratio of requiring additional driving rehabilitation were lower among those with better driving skills (0.17 times), less tension (0.46 times), and less influenced by disability (0.45 times). These results highlight the need to focus on enhancing the driving skills and reducing tension during rehabilitation. Individuals with disabilities who had a driver’s license were significantly influenced by driver response level, type of driver’s license held, and number of assistive devices used than their demographic (excluding sex) or disability characteristics. In this study, the number of assistive devices was treated as a continuous variable to examine whether a greater quantity of devices correlates with increased rehabilitation needs, rather than emphasizing the specific types of devices used. This approach aims to identify whether certain devices may be ineffective or require improvement for particular disabilities, ultimately contributing to the development of more tailored rehabilitation programs.

From the results, variables related to driving, such as those indicating the driver’s ability or type of license previously held, were significant. In particular, the number of assistive devices used while driving was significant, suggesting that the characteristics of assistive devices have a significant impact on the need for additional education for existing license holders. In other words, a higher number of assistive devices in use can be interpreted as having a positive impact on a driver’s safety and technical abilities. This implies a need for driving rehabilitation to enhance the adaptability of each device. As the variety of driving assistive devices expands, training must evolve to address their specialized and diverse requirements. Driving rehabilitation programs should focus not only on technical instruction but also on enhancing drivers’ adaptability to these devices. Tailoring programs to both the features of the devices and the drivers’ unique needs will ensure effective integration into driving practices, improving rehabilitation outcomes. Previous studies have also indicated that training should be provided after all assistive devices have been provided [26–28]. Therefore, training and adaptation to assistive devices are crucial elements in driving training. Lastly, the type of driver’s license also had a significant impact on the need for additional training. Those with a Class 1 license were less likely to need additional training than those with a Class 2 license. This suggests that those with a Class 1 license are more familiar with advanced driving skills and safety rules. These findings suggest that the type of driver’s license should also be considered an important predictor in determining the need for additional driver training.

Our results shed light on predictors of the need for additional driving rehabilitation among individuals with disabilities who hold a driver’s license. These results may serve as evidence for not only developing effective driving training curricula for individuals with disabilities but also for designing measures to facilitate the acquisition of driver licenses among this population to enhance their right to mobility. This study’s findings have several implications. For successful driving training, it is essential to develop professional driving training programs tailored to the individual characteristics and levels of disability of each person. This includes designing appropriate assistive devices, determining the desirable duration of driving training, developing progressive courses that consider the level of tension and difficulty, and selecting the appropriate type of driver’s license. Effective implementation of these professional training programs would require the cultivation of expert personnel to run the programs.

## Conclusion

This study explored factors predicting the need for additional driving rehabilitation training among participants in driving programs. The results revealed that the need for extra training, as determined by driving instructors, was influenced by gender, driving skill, anxiety level, disability impact, use of assistive devices, and type of driver’s license. Women were more likely than men to require additional training (OR: 2.07). Conversely, individuals with better driving skills (OR: 0.17), lower anxiety (OR: 0.46), no disability impact (OR: 0.45), greater use of assistive devices (OR: 0.67), and a Class 1 license (OR: 0.45) were less likely to need further rehabilitation.

These findings highlight key predictors of training needs among drivers with disabilities, providing valuable evidence for developing more effective driving programs and strategies to enhance mobility rights and support driver’s license acquisition for individuals with disabilities.
